# Understanding factors influencing the estimated genetic variance and the distribution of breeding values

**DOI:** 10.3389/fgene.2022.1000228

**Published:** 2022-10-13

**Authors:** Mohammad Ali Nilforooshan, Agustín Ruíz-Flores

**Affiliations:** ^1^ Livestock Improvement Corporation, Hamilton, New Zealand; ^2^ Departamento de Zootecnia, Universidad Autónoma Chapingo, Texcoco, Mexico

**Keywords:** BLUP, breeding values, diagonal elements, genetic variance, heritability, relationship matrix

## Abstract

This study investigated the main factors influencing the genetic variance and the variance of breeding values (EBV). The first is the variance of genetic values in the base population, and the latter is the variance of genetic values in the population under evaluation. These variances are important as improper variances can lead to systematic bias. The inverse of the genetic relationship matrix (**K** ^−1^) and the phenotypic variance are the main factors influencing the genetic variance and heritability (h^2^). These factors and h^2^ are also the main factors influencing the variance of EBVs. Pedigree- and genomic-based relationship matrices (**A** and **G** as **K**) and phenotypes on 599 wheat lines were used. Also, data were simulated, and a hybrid (genomic-pedigree) relationship matrix (**H** as **K**) and phenotypes were used. First, matrix **K** underwent a transformation (K* = *w* **K** + *α* **11**′ + *β* **I**), and the responses in the mean and variation of diag(**K** ^−1^) and offdiag(**K** ^−1^) elements, and genetic variance in the form of h^2^ were recorded. Then, the original **K** was inverted, and matrix **K** ^−1^ underwent the same transformations as **K**, and the responses in the h^2^ estimate and the variance of EBVs in the forms of correlation and regression coefficients with the EBVs estimated based on the original **K** ^−1^ were recorded. In response to weighting **K** by *w*, the estimated genetic variance changed by 1/*w*. We found that *μ*(diag(**K**)) − *μ*(offdiag(**K**)) influences the genetic variance. As such, *α* did not change the genetic variance, and increasing *β* increased the estimated genetic variance. Weighting **K** ^−1^ by *w* was equivalent to weighting **K** by 1/*w*. Using the weighted **K** ^−1^ together with its corresponding h^2^, EBVs remained unchanged, which shows the importance of using variance components that are compatible with the **K** ^−1^. Increasing *β* **I** added to **K** ^−1^ increased the estimated genetic variance, and the effect of *α* **11**′ was minor. We found that larger variation of diag(**K** ^−1^) and higher concentration of offdiag(**K** ^−1^) around the mean (0) are responsible for lower h^2^ estimate and variance of EBVs.

## 1 Introduction

Best linear unbiased prediction (BLUP, Henderson (1975a)) segregates genetic and environmental effects influencing phenotypes, for predicting breeding values. The statistical model, including fixed effects, non-genetic random effects, and random genetic effects, defines how this segregation should be done. Depending on the BLUP type, additive genetic relationships between individuals are modelled *via* **A**, **G**, or **H** matrices (**A** for the pedigree-based BLUP (PBLUP), **G** for genomic BLUP (GBLUP, VanRaden (2008)), and **H** for single-step genomic BLUP (ssGBLUP, Aguilar et al., 2010; Christensen and Lund, 2010), where **H** is a pedigree-genomic hybrid additive relationship matrix). The inverses of these matrices are used in BLUP. Denoting **A**, **G**, and **H** as **K**, BLUP (in its simplest form) is written as:

$$\left[\begin{array}{cc} X^{\prime }X & X^{\prime }Z\\ Z^{\prime }X & Z^{\prime }Z+K^{-1}\lambda \end{array}\right]\left[\begin{array}{c} \hat{b}\\ \hat{u} \end{array}\right]=\left[\begin{array}{c} X^{\prime }y\\ Z^{\prime }y \end{array}\right],$$ (1)

where **X** and **Z** are matrices relating phenotypes to fixed effects and individuals, respectively, **y**, $\hat{b}$ and $\hat{u}$ are the vectors of phenotypes, estimated fixed effects and estimated breeding values (EBV), *λ* is the residual to genetic variance ratio, equal to (1—h^2^)/h^2^, and h^2^ is the heritability of the trait. The greater the genetic variance, the lower the *λ*, and the wider the distribution of EBVs deviated from the mean. Matrix **K** and the phenotypic variance (within and across families) are other factors influencing the genetic variance and the variance of EBVs. The more related the individuals in a population, the less the genetic variation in that population. Genetic variance is a function of *μ*(diag(**K**)) − *μ*(offdiag(**K**)) = *D*_*k*_ (Searle, 1982; Speed and Balding, 2015; Legarra, 2016), and *μ*(**K**) is heavily ((1–1/*n*)%, where *n* is **K**’s dimension) influenced by *μ*(offdiag(**K**)). According to Legarra (2016): *“the genetic variance is the variance of the genetic values of a set of individuals who constitute the reference, or base, population”*, and it is a function of *D*_*k*_. The higher the *D*_*k*_ value, the higher the genetic variance.

For the same population, trait, and model, different **K** may result in different estimates of genetic variance (Legarra, 2016), which can be practically confusing. Also, due to different genetic variances imposed by different **K**, genomic EBVs from GBLUP or ssGBLUP may show a different variance and distribution compared with those from PBLUP, which can be interpreted as bias in the validation of genomic evaluations (Nilforooshan et al., 2010). Tsuruta et al. (2019) studied bias in genomic EBV and reported the incompatibility between **A** and **G**, ignoring inbreeding coefficients in **A** ^−1^, strong selection on a trait (especially when the incompatibility between **A** and **G** is large), inaccurate estimates of unknown parent groups, and using outdated or improper genetic parameters as the main sources of bias in ssGBLUP evaluations. It is interesting to know which properties of **K** elements cause different estimates of genetic variance and distributions of EBVs for different **K**.

Whereas, **G** is built and inverted for GBLUP, **A** ^−1^ and **H** ^−1^ are directly obtained without forming **A** and **H**. Matrix **G** ^−1^ is needed as a part of **H** ^−1^ (Aguilar et al., 2010). In fact, forming and inverting **A** is computationally very intensive (Nilforooshan et al., 2021), even more intensive for **H**. Therefore, knowing about the relationships between the **K** ^−1^ properties and the genetic variance can be as important as the relationships between the **K** properties and the genetic variance. There is little known about the effect of **K** ^−1^ properties on the estimated genetic variance and the distribution of EBVs.

The aim of this study is to find distribution properties of **K** and **K** ^−1^ influencing the estimated h^2^ and the distribution of EBVs. Matrix **K** influences the estimated h^2^ and the distribution of EBVs *via* **K** ^−1^. It defines the way the phenotypic variation is distributed within and across relatives (*e.g.*, full-sib and half-sib families), and the covariance made between phenotypes and genetic effects (equal to the genetic variance). However, the phenotypic variance, individuals on which phenotypes are recorded (*via* **Z**), and other blocks of the mixed model equations, such as those corresponding to additional random effects (if any) also play a role in the proportion of total variance allocated to additive genetic effects, and the distribution of EBVs.

## 2 Materials and methods

### 2.1 Data

#### 2.1.1 Wheat data

The built-in data from R package BGLR (de los Campos and Perez Rodriguez, 2021) was used in this study. The data consist of a pedigree-based additive genetic relationship matrix, genotypes on 1,279 Diversity Array Technology markers, and phenotypes in four environments on 599 CIMMYT wheat lines. Twenty markers were deleted due to having a minor allele frequency less than 0.02. Genotypes and phenotypes (2-year average grain yield) were available for all the lines. Phenotypes in environment one were considered as a single trait to study. Phenotypes were available on all lines and had a *μ* ± sd of 0 ± 1.

#### 2.1.2 Simulated data

Data were simulated to test the hypotheses on more than the wheat data. The R package pedSimulate (Nilforooshan, 2022c) was used for data simulation. The simulation began with a founder population (F0) of 100 males and 100 females randomly mated to each other to produce the next generation. Ten generations were simulated following F0, with no generation overlap. There were 3,144 individuals in the simulated pedigree. The mating ratio was 1:1, and the litter size was 4. Females were selected based on own phenotypes, and in each generation 50% of females were randomly mated to 50% of males. Genotypes were simulated on 5,000 markers. Phenotypes were set to missing for F0, and for 25% of males onwards. Genotypes were retained for generations 8 to 10 (700 individuals), and the rest of genotypes were set to missing. For generations 1 to 10, 10% of sires and 5% of dams were randomly set to missing. Phenotypes were available on 2,554 individuals and had a *μ* ± sd of 48.08 ± 6.48.

### 2.2 Methods

The statistical model was **y** = *μ* + **Zu** + **e**. There was no fixed effect other than the overall mean. Matrix **G** was formed using method 1 of VanRaden (2008): **G** = **WW**′/2*∑p*_*l*_(1 − *p*_*l*_), and then combined with **A** as 0.95**G** + 0.05**A**, where *p*_*l*_ is the marker allele frequency at locus *l*, and **W** is the centered and scaled genotype matrix. The wheat data underwent PBLUP and GBLUP, and the simulated data underwent ssGBLUP (*i.e.*, using **A** ^−1^, **G** ^−1^ and **H** ^−1^ in BLUP (Eq. 1), respectively).

Variance components (genetic and residual variances) were estimated using ASReml statistical software (Gilmour et al., 2015). Breeding values were predicted using the R function solver in the data repository ([Dataset] Nilforooshan, 2022b) to form and solve the mixed model equations. The equation systems were small (600 and 3,145 equations for the wheat and the simulated data, respectively) and the mixed model equations were solved directly.

#### 2.2.1 Properties of **K**

To study the relationships between the properties of **K** and the estimated h^2^ and the distribution of EBVs, matrix **K** underwent the following transformation.

$$K^{*}=wK+\alpha 11^{\prime }+\beta I$$ (2)

Different combinations of *w* (0.9, 1, and 1.1), *α* (–0.05, 0, and 0.05), and *β* (–0.05, 0, and 0.05) were tested on **K**, and the influences on the distributions of diag(**K** ^−1^), offdiag(**K** ^−1^), and the estimated h^2^ were studied. For PBLUP (**K** = **A**), *α* = –0.05 was not tested, as it was leading to negative offdiag(**A**) values. Similarly, *α* = 0 and *β* = –0.05 was not tested on **A**, as it was leading to diagonal values less than 1. One of the main characteristics of **A** as opposed to **G** (and **H**) is having a 1-tailed distribution with the minimum diagonal and off-diagonal values of 1 and 0, respectively.

Coefficients *α* and *β* re-base **K** and diag(**K**), respectively. Coefficient *w* weights **K**, which also changes *μ*(diag(**K**)) − *μ*(offdiag(**K**)), the variation in **K** elements, and the variation in diag(**K**) relative to the variation in offdiag(**K**).

#### 2.2.2 Properties of **K** ^−1^

The original **K** (*w* = 1, *α* = 0, and *β* = 0) was inverted, and then **K** ^−1^ was transformed using Eq. 2 (replacing **K** with **K** ^−1^ in the equation). Different combinations of *w*, *α*, and *β* were tested on **K** ^−1^ (same as those tested on **K**), and the influences on the h^2^ estimate, and the distribution of EBVs were studied. Distribution of EBVs were compared to those using the original **K** ^−1^ (*w* = 1, *α* = 0, and *β* = 0) by regressing the latter to the first, and estimating the Pearson correlation between the two sets of EBV. EBVs were predicted using the h^2^ estimate applying the original **K** ^−1^ *vs.* ${\left(K^{-1}\right)}^{*}$ .

Similar and other transformations are applied to **K** and **K** ^−1^ in the context of ssGBLUP (*e.g.*, Aguilar et al. (2010); Chen et al. (2011); Vitezica et al. (2011); Misztal et al. (2015); MiX99 Development Team (2016); Martini et al. (2018)). However, we emphasize that the transformation in this study (Eq. 2) was for studying the properties of **K** and **K** ^−1^, not fine-tuning them.

## 3 Results and discussion

For the wheat data, **K** corresponds to **A** and **G**, and for the simulated data, **K** corresponds to **H**.

### 3.1 Properties of **K**

In animal populations, matrices **A** and **G** have 1-tailed and 2-tailed distributions skewed toward right, with diagonal and off-diagonal elements centered around 1 and 0, respectively (Simeone et al., 2011; Nilforooshan, 2020). The wheat data showed unusual distributions of **A** and **G** compared to animal data. Also, genotypes for all the markers were 0s and 1s. Distributions of the diagonal and off-diagonal elements of **A** and **G** for the wheat data are presented in Supplementary Figure S1, and the distributions of the diagonal and off-diagonal elements of **A**, **G** and **H** for the simulated data are presented in Supplementary Figure S2. Diagonal elements of **K** had a larger mean and a smaller variation than the off-diagonal elements. Thus, *w* > 1 increased the mean of diag(**K**) further than the mean of offdiag(**K**), and increased the variance of offdiag(**K**) further than the variance of diag(**K**).

Table 1 shows the effect of re-basing and re-scaling **K** *via* *α* and *w*, and re-basing diag(**K**) *via* *β*, on the distribution (*μ* ± sd) of diag(**K** ^−1^). The corresponding results for offdiag(**K** ^−1^) are presented in Table 2. Increasing *w* from 0.9 to 1.1, and *β* from –0.05 to 0.05 reduced both the mean and sd of diag(**K** ^−1^). The reductions by the increase of *β* were more noticeable than the reductions by the increase of *w*. The effect of *α* was marginal. For the wheat data, reduction of *β* to –0.05 had severe consequences on the properties of **K** ^−1^ as in some cases *μ*(diag(**K** ^−1^)) became negative or largely positive, and sd(diag(**K** ^−1^)) and sd(offdiag(**K** ^−1^)) increased tremendously. This numerical instability can be due to **K** getting close to singular by the reduction of *μ*(diag(**K**)) − *μ*(offdiag(**K**)). The consequences were less severe for *w* = 1.1 and more severe for *w* = 0.9. The reason is again due to the change of *μ*(diag(**K**)) − *μ*(offdiag(**K**)) because of *w*. Like **A**, an **A** ^−1^ in a good numerical condition is expected to have a minimum of diagonal values equal to 1 (for an individual with no known parent and progeny). Matrix **A**, which compared to **G** had 3.11 times *μ*(diag(**K**)) − *μ*(offdiag(**K**)), 0.611 times sd(diag(**K**)), and 2.893 times sd(offdiag(**K**)), showed *μ*(diag(**K** ^−1^)) and *μ*(offdiag(**K** ^−1^)) close to those for **G**, 1.750 times sd(diag(**K** ^−1^)), and 1.887 times sd(offdiag(**K** ^−1^)).

**TABLE 1:** The effect of *w*, *α*, and *β* (*w* **K** + *α* **11**′ + *β* **I**) on the distribution of diag(**K** ^**−1**^).

**K** ^1^	*w*	*α*	*β*		
			–0.05	0	0.05
**A**	0.9	0	NA	18.221 ± 52.028	3.653 ± 3.705
		0.05	–5.433 ± 34.812	18.220 ± 52.028	3.653 ± 3.706
	1	0	NA	16.399 ± 46.825	3.478 ± 3.621
		0.05	–3.460 ± 53.157	16.398 ± 46.825	3.477 ± 3.621
	1.1	0	NA	14.908 ± 42.568	3.323 ± 3.542
		0.05	–5.966 ± 117.207	14.908 ± 42.568	3.323 ± 3.542
**G**	0.9	–0.05	87.558 ± 170.569	18.570 ± 29.717	6.437 ± 2.568
		0	539.138 ± 1127.180	18.587 ± 29.733	6.443 ± 2.566
		0.05	105.853 ± 208.039	18.572 ± 29.720	6.438 ± 2.568
	1	–0.05	–16.488 ± 44.850	16.712 ± 26.745	6.092 ± 2.553
		0	–11.828 ± 40.305	16.729 ± 26.759	6.099 ± 2.551
		0.05	–15.978 ± 44.288	16.715 ± 26.748	6.094 ± 2.552
	1.1	–0.05	22.828 ± 58.845	15.193 ± 24.314	5.789 ± 2.533
		0	23.852 ± 59.374	15.208 ± 24.327	5.795 ± 2.531
		0.05	22.988 ± 58.921	15.196 ± 24.316	5.790 ± 2.532
**H**	0.9	–0.05	4.633 ± 2.400	3.119 ± 1.081	2.480 ± 0.676
		0	4.634 ± 2.401	3.120 ± 1.080	2.480 ± 0.676
		0.05	4.633 ± 2.400	3.119 ± 1.081	2.480 ± 0.676
	1	–0.05	3.938 ± 1.918	2.807 ± 0.972	2.276 ± 0.634
		0	3.939 ± 1.919	2.808 ± 0.972	2.276 ± 0.634
		0.05	3.938 ± 1.918	2.807 ± 0.972	2.276 ± 0.634
	1.1	–0.05	3.433 ± 1.601	2.552 ± 0.884	2.103 ± 0.596
		0	3.434 ± 1.601	2.552 ± 0.884	2.103 ± 0.596
		0.05	3.433 ± 1.601	2.552 ± 0.884	2.103 ± 0.596

1 Matrices **A** and **G** correspond to the wheat data, and matrix **H** corresponds to the simulated data.

**TABLE 2:** The effect of *w*, *α*, and *β* (*w* **K** + *α* **11**′ + *β* **I**) on the distribution of offdiag(**K** ^−1^).

**K** ^1^	*w*	*α*	*β*		
			–0.05	0	0.05
**A**	0.9	0	NA	–0.030 ± 2.099	–0.006 ± 0.142
		0.05	0.009 ± 1.342	–0.030 ± 2.099	–0.006 ± 0.142
	1	0	NA	–0.027 ± 1.889	–0.006 ± 0.139
		0.05	0.006 ± 2.134	–0.027 ± 1.889	–0.006 ± 0.139
	1.1	0	NA	–0.025 ± 1.718	–0.006 ± 0.136
		0.05	0.010 ± 6.924	–0.025 ± 1.718	–0.006 ± 0.136
**G**	0.9	–0.05	–0.146 ± 90.017	–0.031 ± 1.111	–0.011 ± 0.156
		0	–0.899 ± 536.710	–0.030 ± 1.112	–0.010 ± 0.156
		0.05	–0.177 ± 107.602	–0.031 ± 1.111	–0.011 ± 0.156
	1	–0.05	0.028 ± 18.065	–0.028 ± 1.000	–0.010 ± 0.151
		0	0.021 ± 15.930	–0.027 ± 1.001	–0.010 ± 0.151
		0.05	0.027 ± 17.785	–0.028 ± 1.000	–0.010 ± 0.151
	1.1	–0.05	–0.038 ± 20.896	–0.025 ± 0.909	–0.010 ± 0.147
		0	–0.039 ± 21.028	–0.025 ± 0.910	–0.009 ± 0.147
		0.05	–0.038 ± 20.913	–0.025 ± 0.909	–0.010 ± 0.147
**H**	0.9	–0.05	--0.001 ± 0.097	–0.001 ± 0.046	–0.001 ± 0.030
		0	–0.001 ± 0.097	–0.001 ± 0.046	–0.001 ± 0.030
		0.05	–0.001 ± 0.097	–0.001 ± 0.046	–0.001 ± 0.030
	1	–0.05	–0.001 ± 0.077	–0.001 ± 0.041	–0.001 ± 0.028
		0	–0.001 ± 0.077	–0.001 ± 0.041	–0.001 ± 0.028
		0.05	–0.001 ± 0.077	–0.001 ± 0.041	–0.001 ± 0.028
	1.1	–0.05	–0.001 ± 0.065	–0.001 ± 0.037	–0.001 ± 0.026
		0	–0.001 ± 0.065	–0.001 ± 0.037	–0.001 ± 0.026
		0.05	–0.001 ± 0.065	–0.001 ± 0.037	–0.001 ± 0.026

1 Matrices **A** and **G** correspond to the wheat data, and matrix **H** corresponds to the simulated data.

The effects of *w*, *α*, and *β* on *μ*(offdiag(**K** ^−1^)), sd(offdiag(**K** ^−1^)), and *μ*(diag(**K** ^−1^)) − *μ*(offdiag(**K** ^−1^)) were similar to those for the mean and sd of diag(**K** ^−1^)). For the simulated data, *μ*(offdiag(**K** ^−1^)) remained –0.001, regardless of *w*, *α*, and *β*. For *α* = 0, *μ*(diag(**K** ^−1^)) − *μ*(offdiag(**K** ^−1^)) was slightly higher compared with *α* = –0.05 and *α* = 0.05, indicating a better numerical condition for **K** ^−1^, since **11**′ is completely singular.

Every covariance matrix **K** can be decomposed to **SQS**, where **S** ^2^ is a diagonal matrix of variances equal to diag(**K**), and **Q** is a correlation matrix. Adding *α* **11**′ + *β* **I** to **K** changes it to S*Q*S*, where ${\left(S^{*}\right)}^{2}=S^{2}+\left(\alpha +\beta \right)I$ . Positive *α* and negative *β* increase the absolute values in Q*. With the correlations getting closer to each other and to 1 or –1, the co-linearity in Q* increases and consequently K* becomes closer to singular. That would cause irregular and unstable distribution of the **K** ^−1^ elements. When **K** reaches singularity and becomes non-positive-definite, it turns non-invertible. Also, *w* **K** is equivalent to multiplying **S** by $\sqrt{w}$ .

The h^2^ was estimated for the simulated data, before and after setting some pedigree information to missing (10% of sires and 5% of dams, for generations 1–10), and the estimates were 0.2910 and 0.2600, respectively. The reduction in the h^2^ is expected as missing pedigree information makes **A** ^−1^ sparser and closer to an identity matrix. Table 3 shows the effects of *w* and *β* applied to **K**, on the h^2^ estimate. There was no h^2^ estimate for **A**, *α* = 0, and *β* = –0.05. The effect of *α* was minor and it did not change the h^2^ estimate rounded to four decimal points. The h^2^ estimate increased slightly by increasing *β* and largely by decreasing *w*. The estimated genetic variance changed 1/*w* times in response to *w* (results not shown). Multiplying **K** by *w*, is equivalent to multiplying the genetic variance by *w*. In response, the estimated genetic variance changed by 1/*w*. Similarly, increasing **S** to S* with a positive *α* + *β* is expected to increase the variances in **K** and reduce the estimated genetic variance and h^2^. However, change of the correlation matrix **Q** to Q* worked in the opposite direction. Consequently, no h^2^ change was observed by changing *α*, and the h^2^ estimate was even increased by increasing *β*. The correlations (**Q**) are expected to increase by increasing *α*. On the other hand, a positive *β* is expected to bring correlations closer to 0, and a negative *β* is expected to deviate correlations from 0.

**TABLE 3:** The effect of *w* and *β* (*w* **K** + *α* **11**′ + *β* **I**) on the heritability estimate.

**K** ^1^	*w*	*β*		
		–0.05	0	0.05
**A**	0.9	0.3533	0.3596	0.3662
	1	0.3302	0.3357	0.3415
	1.1	0.3099	0.3148	0.3199
**G**	0.9	0.7331	0.7610	0.7911
	1	0.7148	0.7413	0.7698
	1.1	0.6974	0.7226	0.7497
**H**	0.9	0.2769	0.2808	0.2848
	1	0.2566	0.2600	0.2634
	1.1	0.2392	0.2421	0.2450

1 Matrices **A** and **G** correspond to the wheat data, and matrix **H** corresponds to the simulated data.

The h^2^ estimates were considerably lower for **A** than for **G** (for the wheat data). The results (Table 3) confirmed previous reports about the role of *D*_*k*_ or alternatively *μ*(diag(**K**)) − *μ*(offdiag(**K**)) in the genetic variance (Searle, 1982; Speed and Balding, 2015; Legarra, 2016). For **A**, *μ*(diag(**K**)) − *μ*(offdiag(**K**)) was considerably higher than for **G**. The higher genetic variance imposed by **A** compared with **G** was compensated by a lower estimate of genetic variance and h^2^. Also, *α* did not make any change to *μ*(diag(**K**)) − *μ*(offdiag(**K**)), neither to the estimates of genetic variance and h^2^. Given the magnitude of *β* in changing *μ*(diag(**K**)) − *μ*(offdiag(**K**)), it played a small role in changing the genetic variance and the h^2^ estimate.

### 3.2 Properties of **K** ^−1^

**FIGURE 1:**
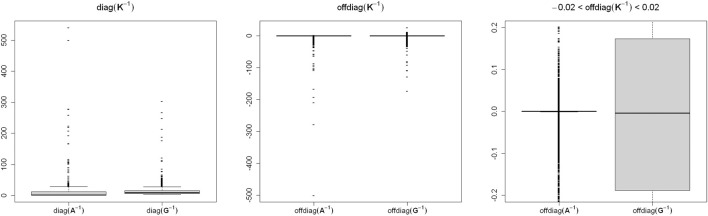
Distribution of **K** ^−1^ elements for the wheat data.

We studied the distributions of **A** ^−1^ and **G** ^−1^ for their large h^2^ difference, and the results are presented in Figure 1. Though the difference between the averages of diag(**A** ^−1^) and diag(**G** ^−1^) was small (Table 1), diag(**A** ^−1^) had a wider range (0.5–540.490 *vs.* 3.324–303.766) compared with diag(**G** ^−1^) (Figure 1). The averages of offdiag(**A** ^−1^) and offdiag(**G** ^−1^) were also similar (Table 2), but offdiag(**A** ^−1^) had a wider range (–500 to 2.936 *vs.* –172.679 to 26.056) compared with offdiag(**G** ^−1^) (Figure 1). Both **A** ^−1^ and **G** ^−1^ had off-diagonal elements concentrated around 0. Whereas, 97.96% of offdiag(**A** ^−1^) were between –0.02 and 0.02, it was 6.37% for offdiag(**G** ^−1^) (Figure 1).

Off-diagonal elements corresponding to max(diag(**A** ^−1^)) showed a lower mean and a higher sd compared with those for max(diag(**G** ^−1^)), –0.904 ± 20.477 *vs.* –0.500 ± 9.064. Diagonal elements corresponding to min(offdiag(**K** ^−1^)) were 590.490 and 500 for **A** ^−1^, and 303.766 and 243.530 for **G** ^−1^, all amongst the largest diagonal elements. Diagonal elements corresponding to max(offdiag(**K** ^−1^)) were moderate to large, 7.666 and 9.089 for **A** ^−1^, and 85.750 and 188.623 for **G** ^−1^.

To determine whether the lower estimates of genetic variance and h^2^ for PBLUP are due to the large ranges of diag(**A** ^−1^) and offdiag(**A** ^−1^), or due to the high concentration of **A** ^−1^ elements (Figure 1), rows and columns of **A** ^−1^ that showed diagonal elements greater than max(diag(**G** ^−1^)) and off-diagonal elements less than min(offdiag(**G** ^−1^)) were discarded. Discarding 10 rows/columns of **A** ^−1^ and their corresponding phenotypes changed the range of diag(**A** ^−1^) to 0.5 and 259.224, and the range of offdiag(**A** ^−1^) to –166.667 and 2.936. The h^2^ estimate changed from 0.3357 (Table 3) to 0.3474, which indicated that the high concentration of **A** ^−1^ elements is mainly responsible for the lower h^2^ estimate for PBLUP compared with that for GBLUP.

The **H** ^−1^ (simulated data) had a range of –1.45 to 2.62 for the off-diagonal elements, and 1 to 6.90 for the diagonal elements. 96.9% of the off-diagonal elements were between –0.02 and 0.02. The distribution of diag(**H** ^−1^) is presented in Supplementary Figure S3. Six rows/columns of **H** ^−1^ corresponding to the largest diag(**H** ^−1^) (ranging from 6.45 to 6.90) were discarded to study the effect of a lower range and variation of diag(**H** ^−1^) on the h^2^ estimate. That reduced max(diag(**H** ^−1^)) to 5.12. Also, *μ*(diag(**H** ^−1^)) − *μ*(offdiag(**H** ^−1^)) reduced from 2.808 to 2.801, but the range of offdiag(**H** ^−1^) did not change. The h^2^ estimate increased from 0.2600 to 0.2637, which confirmed the results from the wheat data, where a lower variation in diag(**K** ^−1^) resulted in a higher h^2^ estimate.

The same transformations applied to **K** (Eq. 2) were applied to the original **K** ^−1^ (i.e., inverted **K** with *w* = 1, *α* = 0, and *β* = 0), and the responses in the h^2^ estimate and EBV distribution were measured. The effects of *w*, *α*, and *β* applied to **K** ^−1^ on the h^2^ estimate are presented in Table 4. The h^2^ estimate increased slightly by increasing *w* and largely by increasing *β*. The effect of *α* was insignificant. The h^2^ estimates were similar for *α* = –0.05 and *α* = 0.05. For the simulated data, *α* = 0 resulted in a slightly higher h^2^ estimate. For the wheat data and *β* = 0.05, *α* = 0 resulted in a slightly higher h^2^ estimate, but it was the opposite when *β* = –0.05. For both **K** and **K** ^−1^, the increase of *β* resulted in a higher h^2^ estimate, and as expected the effect of *w* applied to **K** ^−1^ on the h^2^ estimate was the opposite to that of **K**. The constant *α* almost had no effect on the h^2^ estimate when applied to **K**, and had a small effect on the h^2^ estimate when applied to **K** ^−1^.

**TABLE 4:** The effect of *w*, *α*, and *β* (*w* **K** ^−1^ + *α* **11**′ + *β* **I**) on the heritability estimate.

$K^{-1}$ ^1^	*w*	*α*	*β*		
			–0.05	0	0.05
**A** ^−1^	0.9	–0.05	0.2184	0.3164	0.3793
		0	0.1866	0.3127	0.3843
		0.05	0.2184	0.3164	0.3793
	1	–0.05	0.2513	0.3396	0.3987
		0	0.2108	0.3357	0.4041
		0.05	0.2514	0.3397	0.3987
	1.1	–0.05	0.2807	0.3613	0.4169
		0	0.2296	0.3573	0.4226
		0.05	0.2808	0.3614	0.4170
**G** ^−1^	0.9	–0.05	0.6897	0.7198	0.7437
		0	0.6831	0.7206	0.7458
		0.05	0.6898	0.7199	0.7437
	1	–0.05	0.7150	0.7406	0.7611
		0	0.7102	0.7413	0.7631
		0.05	0.7151	0.7407	0.7612
	1.1	–0.05	0.7365	0.7584	0.7764
		0	0.7328	0.7592	0.7783
		0.05	0.7366	0.7585	0.7765
**H** ^−1^	0.9	–0.05	0.1121	0.2176	0.2829
		0	0.1433	0.2402	0.3004
		0.05	0.1122	0.2176	0.2830
	1	–0.05	0.1402	0.2360	0.2988
		0	0.1725	0.2600	0.3175
		0.05	0.1403	0.2361	0.2988
	1.1	–0.05	0.1650	0.2536	0.3139
		0	0.1982	0.2788	0.3337
		0.05	0.1651	0.2537	0.3140

1 Matrices **A** and **G** correspond to the wheat data, and matrix **H** corresponds to the simulated data.

Correlation coefficients between EBV from different **K** ^−1^ and the original **K** ^−1^ (*w* = 1, *α* = 0, and *β* = 0) are presented in Table 5. The h^2^ estimate from the original **K** ^−1^ was used for the EBV prediction in all the analyses, (1) to be able to distinguish between the effects of **K** ^−1^ and h^2^ used in the mixed model equation, and (2) estimation of variance components is computationally intensive, and in routine genetic evaluations, usually, it is not affordable to update them regularly. Deviation of *w* from 1, and *α* and *β* from 0 reduced the EBV correlations. Differences in the correlations due to *w* and *β* were minor. Differences were also minor for *α* and the wheat data. However, deviation of *α* from 0 substantially reduced the correlations for the simulated data, which might be an indication of **K** ^−1^ further inflicted by singularity than for the wheat data.

**TABLE 5:** The effect of *w*, *α*, and *β* (*w* **K** ^−1^ + *α* **11**′ + *β* **I**) on the correlation of predicted breeding values with those using the original **K** ^−1^ (*w* = 1, *α* = 0, and *β* = 0). The same heritability (based on the original **K** ^−1^) was used in all the analyses.

$K^{-1}$ ^1^	*w*	*α*	*β*		
			–0.05	0	0.05
**A** ^−1^	0.9	–0.05	0.9925	0.9926	0.9922
		0	0.9789	0.9998	0.9956
		0.05	0.9926	0.9926	0.9923
	1	–0.05	0.9921	0.9925	0.9926
		0	0.9745	1.0000	0.9963
		0.05	0.9921	0.9925	0.9926
	1.1	–0.05	0.9913	0.9921	0.9925
		0	0.9691	0.9998	0.9967
		0.05	0.9914	0.9922	0.9925
**G** ^−1^	0.9	–0.05	0.9987	0.9986	0.9985
		0	0.9997	0.9998	0.9997
		0.05	0.9987	0.9986	0.9986
	1	–0.05	0.9987	0.9987	0.9987
		0	0.9998	1.0000	0.9999
		0.05	0.9987	0.9988	0.9988
	1.1	–0.05	0.9985	0.9985	0.9986
		0	0.9996	0.9998	0.9998
		0.05	0.9985	0.9986	0.9986
**H** ^−1^	0.9	–0.05	0.2691	0.2650	0.2610
		0	0.9980	0.9998	0.9970
		0.05	0.2773	0.2736	0.2699
	1	–0.05	0.2712	0.2712	0.2634
		0	0.9978	1.0000	0.9982
		0.05	0.2803	0.2768	0.2733
	1.1	–0.05	0.2729	0.2692	0.2654
		0	0.9975	0.9999	0.9989
		0.05	0.2829	0.2796	0.2763

1 Matrices **A** and **G** correspond to the wheat data, and matrix **H** corresponds to the simulated data.

Regression coefficients of the EBVs using different **K** ^−1^ on the EBVs using the original **K** ^−1^ (*w* = 1, *α* = 0, and *β* = 0) are presented in Table 6. The h^2^ estimate using the original **K** ^−1^ was used for the EBV prediction in all the analyses. Increasing *w* and *β* and deviation of *α* from 0 reduced the regression coefficient, as a result of inflated evaluations. Where the regression coefficient is greater than the correlation coefficient, it means that the variance of EBVs has increased, and *vice versa*. Generally, decreasing *w* and *β* resulted in increasing the variance of EBVs. Unless **K** ^−1^ has not inflicted by the singularity of *α* **11**′ (*e.g.*, the simulated data), the role of *α* on changing the variance of EBVs was marginal.

**TABLE 6:** The effect of *w*, *α*, and *β* (*w* **K** ^−1^ + *α* **11**′ + *β* **I**) on the regression coefficient of predicted breeding values on those using the original **K** ^−1^ (*w* = 1, *α* = 0, and *β* = 0). The same heritability (based on the original **K** ^−1^) was used in all the analyses.

$K^{-1}$ ^1^	*w*	*α*	*β*		
			–0.05	0	0.05
**A** ^−1^	0.9	–0.05	1.0685	0.9975	0.9359
		0	1.0412	1.0335	0.9453
		0.05	1.0686	0.9975	0.9359
	1	–0.05	1.0292	0.9624	0.9044
		0	0.9974	1.0000	0.9147
		0.05	1.0293	0.9625	0.9044
	1.1	–0.05	0.9934	0.9304	0.8755
		0	0.9567	0.9693	0.8866
		0.05	0.9935	0.9304	0.8755
**G** ^−1^	0.9	–0.05	1.0296	1.0154	1.0016
		0	1.0400	1.0225	1.0069
		0.05	1.0297	1.0155	1.0017
	1	–0.05	1.0063	0.9926	0.9793
		0	1.0168	1.0000	0.9850
		0.05	1.0064	0.9927	0.9794
	1.1	–0.05	0.9848	0.9715	0.9587
		0	0.9953	0.9791	0.9646
		0.05	0.9849	0.9716	0.9588
**H** ^−1^	0.9	–0.05	0.1759	0.1558	0.1400
		0	1.2041	0.9957	0.8434
		0.05	0.1807	0.1603	0.1443
	1	–0.05	0.1646	0.1466	0.1324
		0	1.2023	1.0000	0.8508
		0.05	0.1696	0.1514	0.1369
	1.1	–0.05	0.1549	0.1387	0.1257
		0	1.1974	1.0012	0.8554
		0.05	0.1601	0.1436	0.1304

1 Matrices **A** and **G** correspond to the wheat data, and matrix **H** corresponds to the simulated data.

For different *w*, *α* = 0, and *β* = 0, EBVs remained unchanged using the h^2^ estimate from the same **K** ^−1^ rather than the original **K** ^−1^ ([Dataset] Nilforooshan, 2022b). This means that weighting **K** ^−1^ by a scalar, that scalar is captured by the variance component estimation, and using the estimated variance components from the same **K** ^−1^, EBVs remain unchanged. Matrix **K** ^−1^ can be decomposed to **S** ^−1^ **Q** ^−1^ **S** ^−1^. Multiplying *w* to **K** ^−1^ corresponds to dividing **S** by $\sqrt{w}$ . Adding *α* **11**′ + *β* **I** to **K** ^−1^ changes it to ${\left(S^{-1}\right)}^{*}{\left(Q^{-1}\right)}^{*}{\left(S^{-1}\right)}^{*}$ , where ${\left(S^{-2}\right)}^{*}=S^{-2}+\left(\alpha +\beta \right)I$ .

### 3.3 Factors defining the elements of **K** and **K** ^−1^

Different factors define the elements of **A** and **G** (similarly **H**, in which the genomic information in **G** is propagated to non-genotyped individuals). Diagonal elements of **A** are twice the probability of two random gametes from an individual carrying identical by descent alleles, and offdiag(**A**) are equal to the numerator of the coefficients of relationship between pairs of individuals (Wright, 1922). Diagonal elements of **G** increase as an individual’s homozygosity rate increases, further by homozygosity for rare alleles (VanRaden, 2008). Off-diagonal elements of **G** increase as the shared homozygosity rate (*i.e.*, homozygosity at the same loci) between pairs of individuals increases, further by shared homozygosity for rare alleles. Following method 1 of VanRaden (2008), diagonal element of **G** for individual *i*, and the off-diagonal element of **G** between individuals *i* and *j* equal:

$$G_{ii}=\overset{n}{\underset{l=1}{\sum }}{\left(M_{il}-2p_{l}+1\right)}^{2}/2\overset{n}{\underset{l=1}{\sum }}p_{l}\left(1-p_{l}\right),$$ (3)

$$G_{ij}=\overset{n}{\underset{l=1}{\sum }}\left(M_{il}-2p_{l}+1\right)\left(M_{jl}-2p_{l}+1\right)/2\overset{n}{\underset{l=1}{\sum }}p_{l}\left(1-p_{l}\right),$$ (4)

Where *M*_*ij*_ is the genotype (coded as –1, 0, 1) of individual *i* at locus *l*, and *n* is the total number of genotype markers.

Diagonal elements of **A** ^−1^ increase by the number of individual’s progeny, known mates, and known parents (Henderson, 1975b). Considering no parent-progeny mating for an easier explanation, positive offdiag(**A** ^−1^) are those between mates, which increase as the number of their progeny increases, and negative offdiag(**A** ^−1^) are those between parent and progeny. Individual *i*’s inbreeding coefficient adds to the elements of **A** ^−1^ corresponding to its progeny and to *A* ^*ii*^ (the diagonal element of **A** ^−1^ for individual *i*), and deducts from the elements corresponding to its mates (Nilforooshan, 2022a). Little is known about factors influencing elements of **G** ^−1^. We found a correlation of 0.091 between diag(**A**) and diag(**A** ^−1^), and –0.194 between diag(**G**) and diag(**G** ^−1^).

## 4 Conclusion

Different **K** and **K** ^−1^ result in different h^2^ estimates and distributions of EBVs. We studied some distribution properties of **K** and **K** ^−1^ elements to learn about the properties of the two matrices influencing the h^2^ estimate and the distribution of EBVs. Higher phenotypic variance and h^2^ result in higher genetic variance and the variance of EBVs. Furthermore, the distribution of **K** and **K** ^−1^ elements are important as they directly and indirectly (*via* the estimated h^2^) influence the genetic variance and the variance of EBVs. Matrix **K**, which defines the genetic relatedness among individuals and the amount of inbreeding, underwent a combination of three transformations, each with three levels. Adding *α* **11**′ to **K** is equivalent to re-basing the base population to a former generation with a positive *α* (Chen et al., 2011; Vitezica et al., 2011), which is applied in the context of ssGBLUP for improving the compatibility between **G** and **A**. Note that **11**′ is completely singin the ular, and an *α* largely deviated from 0 would bring **K** closer to singular. Similarly, adding *β* **I** to **K** with a negative *β* brings **K** closer to singular. Therefore, the conclusions of this study exclude odd observations that might occur because of **K** or **K** ^−1^ becoming singular or nearly singular. Though eigenvalues of **K** are direct indicators of **K**’s numerical condition, negative or high *μ*(diag(**K** ^−1^)), and/or very large sd(diag(**K** ^−1^)) and sd(offdiag(**K** ^−1^)) are indirect indicators of **K**’s ill numerical condition.

Depending on the sign of *β*, *μ*(diag(**K**)) − *μ*(offdiag(**K**)) or *D*_*k*_ is changed. A positive *β* means adding to inbreeding coefficients while keeping genetic covariances (i.e., offdiag(**K**)) unchanged. It also means adding unstructured variance to the structured genetic variance, which resulted in the increase of the genetic variance, the h^2^ estimate, and the variance of EBVs. In inbred populations, offdiag(**K**) increase further than diag(**K**) by inbreeding. As a result of the reduced *D*_*k*_, the genetic variance, and the variance of EBVs are expected to reduce. Weighting **K** by *w*, both the genetic variance and *D*_*k*_ are weighted. This weight is captured by the variance component estimation and in response to the genetic variance multiplied by *w*, 1/*w* of the genetic variance is estimated, which is equivalent to changing h^2^ from 1/(1 + *λ*) to *w*/(*w* + *λ*). Similarly, weighting **K** ^−1^ by *w*, *w* times the genetic variance is estimated, which is equivalent to changing h^2^ to 1/(1 + *w* *λ*). Applying the weighted **K** ^−1^ and its corresponding variance components in the form of h^2^, EBVs remained unchanged. This shows the importance of using variance components corresponding to the **K** ^−1^ that is used in BLUP. Using improper or outdated variances is an important source of bias in genetic evaluations (Tsuruta et al., 2019). Variance component estimates need to be updated regularly, and the use of variance components estimated based on a type of **K** ^−1^ (*e.g.*, **A** ^−1^) should be avoided in BLUP using other **K** ^−1^ (e.g., **G** ^−1^ and **H** ^−1^). This study showed very different h^2^ estimates for the same population and trait, based on different **K** ^−1^ (between **A** ^−1^ and **G** ^−1^ for the wheat data, between transformed **K**, and between transformed **K** ^−1^).

In agreement with previous studies, we found *D*_*k*_ as a distribution parameter of **K** influencing the genetic variance. We studied *μ*(diag(**K**)) − *μ*(offdiag(**K**)) instead of *D*_*k*_, however for large matrices like **K** (individual × individual), *μ*(**K**) is heavily defined by *μ*(offdiag(**K**)). Matrix **K** ^−1^ underwent the same transformations as matrix **K**. Coefficient *α* re-bases the matrix, coefficient *β* re-bases the diagonal elements, and coefficient *w* inversely weights the genetic variance and changes the mean and the variance of the matrix, further for the diagonal elements, since those have larger mean and variance. We found that, *w* and *β* applied to **K** ^−1^ influence the h^2^ estimate and the distribution of EBVs. The effect of *α* was marginal for both **K** and **K** ^−1^, unless it inflicts them with singularity. We also found that lower variance of diag(**K** ^−1^) can result in higher h^2^ and thus greater variance of EBVs. For the off-diagonal elements of **K** ^−1^, more important than the variation of the elements, was how they are concentrated around their mean. For example, the h^2^ estimate by **G** ^−1^ was considerably greater than for **A** ^−1^, for the wheat data. Whereas the off-diagonal elements of **G** ^−1^ showed a smaller range and variation, those were more widely centered around 0 than the off-diagonal elements of **A** ^−1^.

## Data Availability

Publicly available datasets were analyzed in this study. The data can be found here: https://cran.r-project.org/package=BGLR, https://doi.org/10.17632/zd3xx54j62.3.
