# Supplementary material for: Understanding factors influencing the estimated genetic variance and the distribution of breeding values
Source: Front Genet. 2022 Oct 13;13:1000228. doi: 10.3389/fgene.2022.1000228 (PMC9606665; doi:10.3389/fgene.2022.1000228)
Supplement: Supplementary file 1 [file Image1.PDF]

Supplementary Material

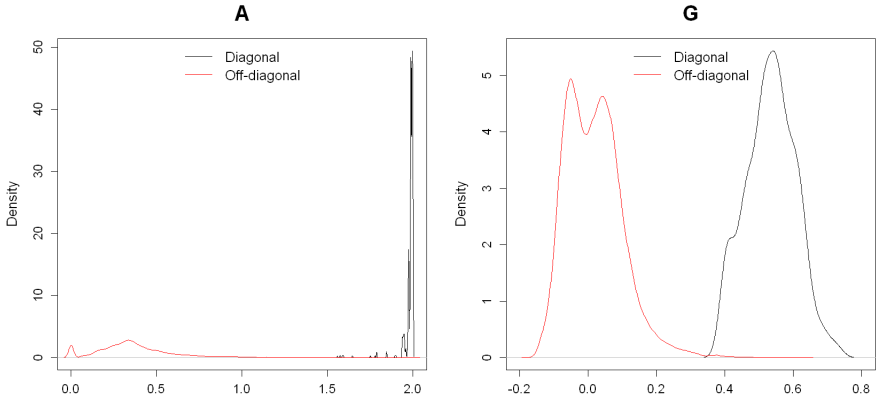

Figure S1. Distributions of **A** and **G** elements for the wheat data (**A** and **G** are the pedigree- and genomic-based relationship matrices).

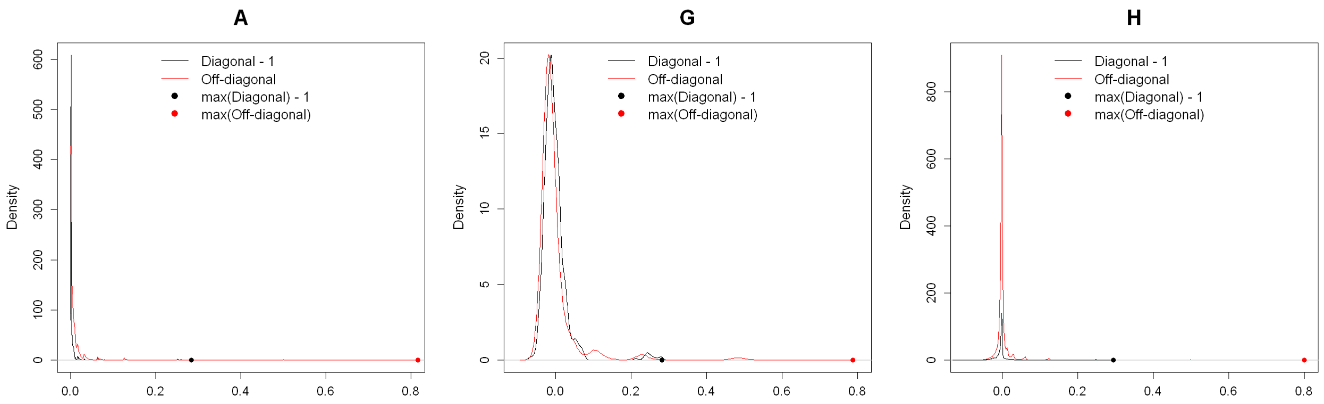

Figure S2. Distributions of **A**, **G** and **H** elements for the simulated data (**A**, **G** and **H** are the pedigree-based, genomic-based, and hybrid relationship matrices).

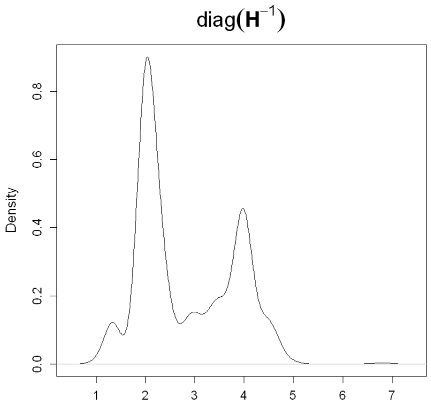

Figure S3. Distribution of the diagonal elements of $\mathbf{H}^{-1}$ for the simulated data (**H** is the pedigree-genomic hybrid relationship matrix).
